# *Echinococcus* in wild canids in Québec (Canada) and Maine (USA)

**DOI:** 10.1371/journal.pntd.0006712

**Published:** 2018-08-20

**Authors:** Janna M. Schurer, Emilie Bouchard, Ann Bryant, Sarah Revell, Grace Chavis, Anne Lichtenwalner, Emily J. Jenkins

**Affiliations:** 1 Department of Veterinary Microbiology, University of Saskatchewan, Saskatoon, Saskatchewan, Canada; 2 School of Food and Agriculture, University of Maine, Orono, Maine, United States of America; 3 Cooperative Extension, University of Maine, Orono, Maine, United States of America; Negrar Hospital, ITALY

## Abstract

Zoonotic *Echinococcus* spp. cestodes (*E*. *canadensis* and *E*. *multilocularis*) infect domestic animals, wildlife, and people in regions of Canada and the USA. We recovered and quantified *Echinococcus* spp. cestodes from 22 of 307 intestinal tracts of wild canids (23 wolves, 100 coyotes, 184 red and arctic foxes) in the state of Maine and the province of Québec. We identified the species and genotypes of three *Echinococcus* spp. cestodes per infected animal by sequencing mitochondrial DNA at two loci. We further confirmed the absence of *E*. *multilocularis* by extracting DNA from pools of all cestodes from each animal and running a duplex PCR capable of distinguishing the two species. We detected *E*. *canadensis* (G8 and G10), but not *E*. *multilocularis*, which is emerging as an important human and animal health concern in adjacent regions. Prevalence and median intensity of *E*. *canadensis* was higher in wolves (35%, 460) than coyotes (14%, 358). This parasite has historically been absent in Atlantic regions of North America, where suitable intermediate hosts, but not wolves, are present. Our study suggests that coyotes are serving as sylvatic definitive hosts for *E*. *canadensis* in Atlantic regions, and this may facilitate eastward range expansion of *E*. *canadensis* in the USA and Canada. As well, compared to wolves, coyotes are more likely to contaminate urban green spaces and peri-urban environments with zoonotic parasites.

## Author summary

Echinococcosis is a zoonosis caused by ingestion of tapeworm eggs in feces of wild or domestic canids (e.g. foxes, wolves, coyotes, and dogs). In North America, the number of new human echinococcosis cases reported annually is low; however, recent reports of these parasites in unusual presentations, in new locations, and in wildlife near urban areas have caused renewed interest by veterinary and human health professionals. In a cross-border collaboration, we examined the intestines of wolves (*Canis lupus*), coyotes (*C*. *latrans*) and foxes (*Vulpes vulpes*, *V*. *lagopus*) trapped in Québec (Canada) and neighboring Maine (USA), using genetic tools to identify *Echinococcus* tapeworms. We did not detect *E*. *multilocularis*, a serious threat to human health that has recently emerged in southern Ontario. We did identify *E*. *canadensis* in wolves and coyotes, in both Quebec and Maine. The presence of this parasite in coyotes is especially concerning because coyotes are more likely to come into close proximity with human communities. This information is relevant to veterinarians who should promote regular fecal examination and/or deworming of high risk dogs (dogs that scavenge or hunt cervids, such as moose), to physicians who might encounter this relatively rare disease, and to public health agencies who should promote surveillance and develop precautions for high risk people.

## Introduction

**Fig 1:**
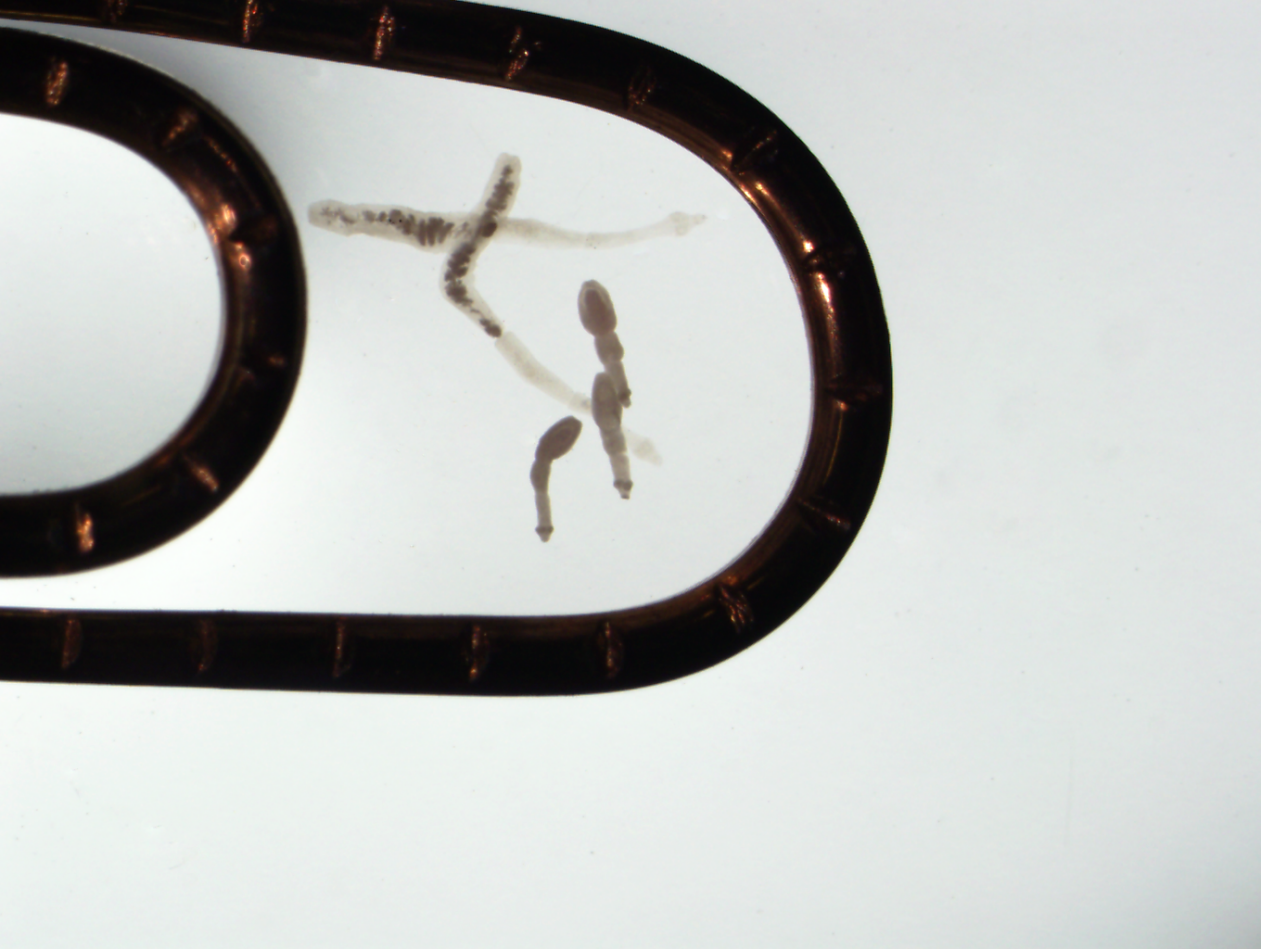
*Echinococcus canadensis* (upper two) and *E*. *multilocularis* (bottom three) adult cestodes (Credit: Brent Wagner).

Recent findings suggest that two species of *Echinococcus* are emerging as threats to public health in regions of Canada and the USA [1]. These include range expansion of *E*. *multilocularis* at its western and eastern distributional limits in Canada, and the novel identification of *E*. *canadensis* G8 in moose in Maine [2,3]. The *Echinococcus* genus is a group of cestodes maintained by specific predator-prey host assemblages in a wide range of geographic and climatic locales around the world [4]. Generally, wild or domestic canids act as definitive hosts, harboring adult cestodes in the small intestines (Fig 1) that shed infectious eggs via feces into the environment. Intermediate hosts are specific to each *Echinococcus* species but can include sheep/goats, cattle, swine, cervids, horses, camels, as well as various rodent species. These hosts develop fluid-filled cysts containing the larval protoscolices that are infective to carnivore definitive hosts when ingested. People are infected when they accidentally ingest *Echinococcus* eggs of canid fecal origin that contaminate food, water, or the environment [5]. Over time, the ingested eggs develop into space-occupying cysts in organs such as the liver and lungs. The prognosis and medical costs for such patients is highly variable, and depend largely on the parasite species ingested, the immune status of the person, how early the infection is detected, and the level of access to medical services [5].

The species of *Echinococcus* present in Canada are *E*. *canadensis* (genotypes G8 and G10), also known as the cervid or sylvatic strain, and various strains of *E*. *multilocularis*[6,7]. In the USA, both of these sylvatic species are present, as well as one other—*E*. *granulosus* sensu stricto, also known as the domestic or sheep strain [6]. *Echinococcus canadensis* circulates in cervid-canid host assemblages (e.g. moose [*Alces alces*]-wolf [*Canis lupus*]), and is distributed across Canada, except for the high Arctic Islands and the Atlantic provinces [8]. The distribution of *E*. *canadensis* is not as well characterized in the USA, but G8 and/or G10 genotypes have been reported in Alaska, Washington, Minnesota, and, recently, Maine [6]. *Echinococcus multilocularis* was traditionally considered endemic in two distinct regions: the Northern Tundra Zone (NTZ) in northwestern Alaska and the Canadian Arctic, and the North Central Region (NCR) in northcentral USA and southcentral Canada [5]. However, these two regions are no longer discontinuous and the parasite has a broader host and geographic distribution than previously suspected [9].

The hypothesis that human risk of echinococcosis is increasing in North America is supported by recent reports documenting *E*. *canadensis and E*. *multilocularis* outside traditional geographic and host boundaries. In 2014, *E*. *canadensis* G8 was identified for the first time in the state of Maine in a sylvatic moose population [2]. At the same time, *E*. *multilocularis* was reported in Canadian wolves and coyotes outside of the NTZ and NCR [3,10]. The first canine case of alveolar echinococcosis in North America was detected in 2009, and subsequently at least 15 cases have been detected across most of western Canada and Ontario [11,12]. Human cases of echinococcosis are rare in Canada, and infection origin is often not determined. Between 2002 and 2011, the Canadian Institute for Health Information recorded 251 cases of echinococcosis (species undetermined), 48 cases of cystic echinococcosis and 16 cases of alveolar echinococcosis, but did not report whether these cases were domestically acquired [13]. One of the five alveolar echinococcosis cases diagnosed in Alberta between 2013 and 2018 has so far proven autochthonous, prompting renewed public health attention to this parasite [14] (S. Houston pers comm.). A key barrier to evaluating the importance of these findings is the lack of baseline data in eastern Canada and the USA. Therefore, we aimed to inform future public health threat assessments by (i) identifying the definitive host(s) of *E*. *canadensis* in Maine, and (ii) determining if *E*. *multilocularis* had spread from Ontario to Québec, and (iii) developing baseline data on *Echinococcus* distribution and wildlife hosts in Québec. The province of Québec was chosen because it borders on Maine, is adjacent to Ontario where *E*. *multilocularis* is rapidly emerging as a concern in canids [3], and because surveillance data on Québec canids dates to the 1980s [15].

## Methods

Hunters and trappers in Québec and the Maine Department of Inland Fisheries and Wildlife provided carcasses of wild wolves, coyotes and foxes (red and arctic) that were harvested for non-research purposes over the winter of 2016/17. Intestinal tracts were removed and stored at -80˚C for at least 5 days to inactivate infectious *Echinococcus* eggs as per World Health Organization standards [5], and at -20˚C otherwise. *Echinococcus* spp. cestodes were collected from small intestines by the scraping, counting, and filtration method [16] after thawing the tracts at room temperature. Average infection intensity was estimated by suspending all of these cestodes in 100 mL of dH_2_O and counting the scolices in two 10% aliquots pipetted into grid-lined petri dishes examined under a dissecting microscope. Cestodes were stored in 90% ethanol at room temperature prior to molecular analysis.

To identify *Echinococcus* species and genotypes, we randomly selected three intact cestodes from each infected canid and extracted DNA using a thermocycler tissue lysis technique [17]. We conducted Polymerase Chain Reaction (PCR) with two primer sets capable of differentiating genotypes: nicotinamide adenosine dinucleotide dehydrogenase subunit 1 (ND1) and cytochrome *c* oxidase subunit 1 (CO1), to amplify two separate regions of mitochondrial DNA [18,19]. PCR products were resolved by electrophoresis on a 1.5% agarose gel. Single cestode PCR products were purified using the QIAquick PCR Purification Kit (Qiagen Inc., Valencia, California, USA), and sequenced (Macrogen Inc., Seoul, Korea). Forward and reverse sequences were trimmed, aligned, and then identified using the BLASTn tool to compare the similarity of sample sequences to reference sequences in the nucleotide database of GenBank [20]. Similar to Santa et al [7], we then pooled all the *Echinococcus* spp. cestodes remaining from each infected animal and extracted DNA from these pools (up to 100 mg per reaction) using the QIAamp Fast DNA Stool Mini Kit (Qiagen Inc., Valencia, California, USA). We modified the manufacturer protocol to increase tissue disruption by shaking the cestodes at 4 m/s for 20s in lysis matrix tubes containing a garnet matrix and ¼ inch spherical beads (MP Biomedicals; Solon, Ohio, USA) in the initial step and eluting 100 μL of DNA in the final step. To detect *E*. *granulosus/canadensis* and *E*. *multilocularis* in these pooled samples we conducted a duplex PCR with two gene targets: ND1 and the small subunit of ribosomal of RNA (rrnS) [21].

All data were analyzed using R version 3.4.3 [22]. Host species were compared to infection status by a 2-sided Fisher’s exact test, using a significance threshold of 0.05. The Mann-Whitney U-Test was used to evaluate infection intensity differences among canid host species. The distribution of infected and uninfected canids was mapped by entering the geographic coordinates (latitude, longitude) of trap sites into ArcGIS (v10.2.2; Esri, Redlands, CA, USA).

According to Canadian Council on Animal Care guidelines, this research was exempt from Animal Research Ethic Board review in Canada because all tissues were sourced from animals harvested for non-research purposes. This research was found to be exempt from Institutional Animal Care and Use Committee approval in the USA because all tissues were sourced from animals harvested as part of an existing coyote management program.

## Results

**Fig 2:**
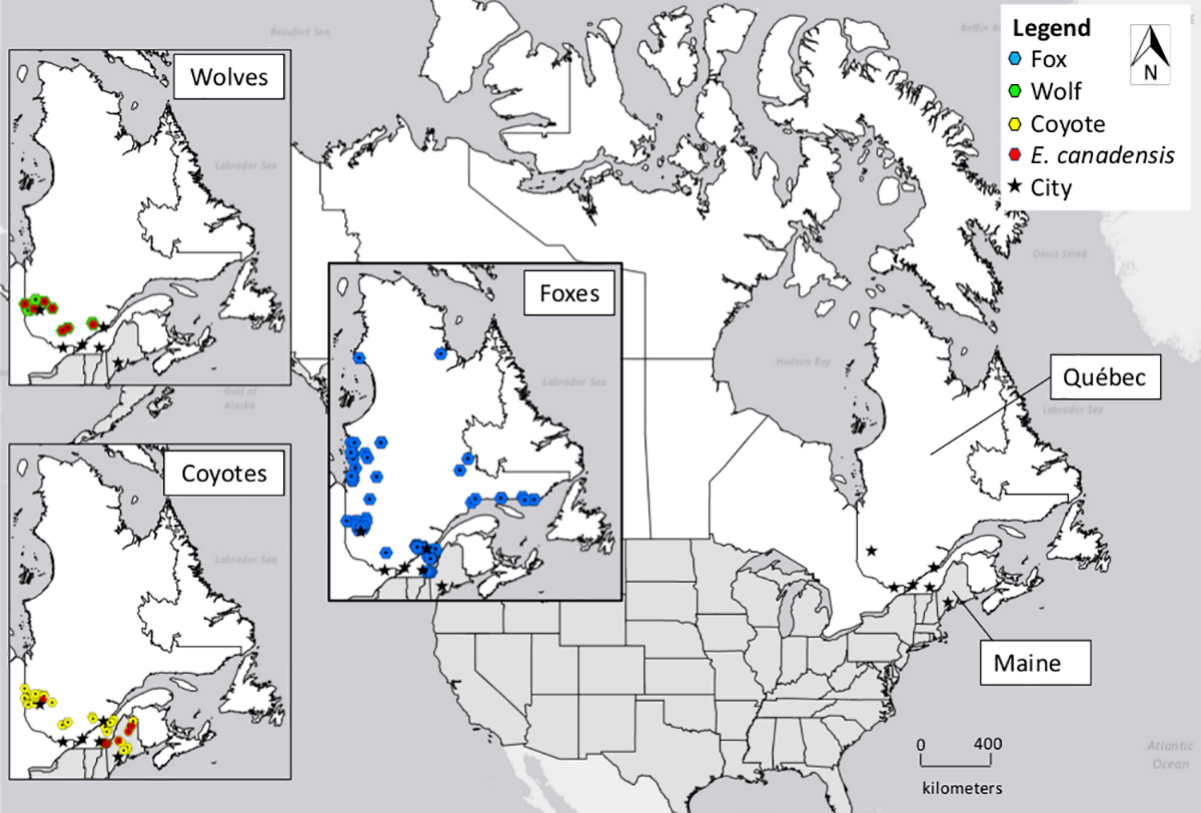
Sampling distribution and *Echinococcus* infection status of wild canids (N = 307) from Québec, Canada and Maine, USA (Source: ArcGIS v10.2.2).

We examined the intestinal tracts of 23 wolves, 77 coyotes, 181 red foxes, and 3 arctic foxes submitted by Québec trappers, and 23 coyotes submitted from Maine (Total N = 307; Fig 2). Altogether, *Echinococcus* infection prevalence was significantly higher in wolves (35% of 23, 95% CI = 16–57) versus coyotes (14% of 100, 95% CI = 8–22; X^2^ (df1) = 4.18, p-value = 0.032; Table 1). The prevalence difference in coyotes from Maine (22% of 23, 95% CI = 7–44) versus Québec (12% of 77, 95% CI = 5–21) was not significant (X^2^ (df1) = 0.77, p-value = 0.30). No *Echinococcus* spp. cestodes were detected in red or arctic foxes (0% of 184, 95% CI = 0–2). The overall median infection intensity was 358 ± 1508 cestodes/canid (range: 5–6038 cestodes/canid), with no significant difference between wolves (460 ± 1110 cestodes/wolf) and coyotes (358 ± 1750 cestodes/coyote; p-value = 0.80).

**Table 1:** Prevalence of *Echinococcus* species and genotypes in wild canids from Québec, Canada and Maine, USA.

	Wolves (N = 23)		Coyotes (N = 100)		Red /Arctic Foxes (N = 184)		Overall (N = 307)	
	n	%	n	%	N	%	n	%
*E*. *canadensis*								
G8 only	5	22	6	6	0	0	11	3.6
G10 only	1	4.3	2	2	0	0	3	1.0
G8 & G10	2	8.7	6	6	0	0	8	2.6
*E*. *canadensis* (total)	8	35	14	14	0	0	22	7.2

In most cases, we obtained mitochondrial DNA sequences for three *Echinococcus* spp. cestodes per host. No *E*. *multilocularis* was detected. Overall, single *E*. *canadensis* G8 infections were most common (11/22, 50%), followed by mixed *E*. *canadensis* G8/G10 infections (8/22, 36%) and single *E*. *canadensis* G10 infections (3/22, 13%). In Maine, we observed three canids with G8 infections and two with mixed G8/G10 infections. Our PCR duplex of pooled *Echinococcus* samples confirmed the results of the single cestode analysis, and detected DNA of *E*. *canadensis* but not *E*. *multilocularis*. Single cestode DNA sequences were 98–100% similar to reference sequences in the nucleotide database of GenBank. Sample sequences were most similar to complete G8 and G10 genome sequences from moose in the USA (accession number: AB235848) and in Finland (accession number: AB745463), respectively [23]. There were no disagreements in species or genotype identity obtained by CO1 versus ND1 sequence data; DNA sequences from pooled samples were not sequenced to determine genotype(s). High quality sequences of suitable length were trimmed (COI- 374 bps, ND1-485 bps), submitted to Genbank, and assigned accession numbers (G8: MG561268-72, MG574822-7 MG582994-MG583003; G10:MG583004-19).

Infected canids were distributed from east to west across Québec and Maine with no cases observed in foxes from northern regions of Québec or in coyotes from southern coastal regions of Maine (Fig 2). Although most infected animals were captured in rural/remote areas, infected wolves and coyotes were observed close to urban centers (e.g. Sherbrooke and Val d’Or, Québec). Infected wolves were observed in each locale sampled.

## Discussion

This study confirms the presence of *E*. *canadensis* (G8 and G10 strains) in wild canids on both sides of the Canada-USA border in eastern North America. In Maine, where this parasite was thought to be absent due to the historical absence of wolves, we identified coyotes as a sylvatic definitive host, and identified a previously unreported genotype to the area (G10). Previous wild canid studies have reported the G8 genotype in wolves from Canada, Russia, and the USA [1,24]. The G10 genotype has been reported in wolves from Canada, Russia, Estonia, Mongolia, and the USA, as well as in red foxes and coyotes in Canada [1,7,24]. A few infection clusters occurred in close proximity to urban centers in Québec, indicating that there is a need for human health professionals and veterinarians to collaboratively increase awareness about this parasite. This is especially important in light of recent human cystic echinococcosis cases caused by the G8 strain in QC (pers. comm. C. Yansouni, McGill University Health Centre, June 1, 2018). We did not detect *E*. *multilocularis* in wild canids, despite widespread sampling and recent detection in canids from the neighbouring province of Ontario. We also did not detect the livestock variant, *E*. *granulosus*, which is endemic to certain states in the USA.

Our finding of *E*. *canadensis* G8 and G10 in Maine coyotes builds upon the first published report of *E*. *canadensis* G8 in 39% of 54 moose sampled in 2014 as part of a lungworm survey, by extending the sampling area farther south [2]. Infected coyotes were only detected in the north and west of the state, suggesting that infected wildlife is likely crossing the Canada-USA border. Public health messaging in Maine should emphasize the importance of prophylactic cestocidal treatment of domestic dogs with access to cervid carcasses, such as those used for hunting, as dogs can act as bridging hosts between wildlife and people [25]. Livestock producers should be aware that *E*. *canadensis* is a risk for captive cervids [26], but not domestic livestock. A comprehensive survey of domestic and wildlife hosts along the southern and western state limits would complete this initial assessment of *Echinococcus* prevalence in Maine, and allow for a more informed assessment of public health risk. As well, it might identify definitive and intermediate hosts other than coyotes and moose, as *E*. *canadensis* has previously been detected in a range of ungulate hosts in Canada, including cervids and muskoxen [8].

Within Québec, we extended the known distribution of *E*. *canadensis* infected wolves beyond the last published report (La Verendrye Provincial Game Reserve) in the 1980s, to include wolves trapped near Québec City towards the east and near Val d’Or towards the northwest [15]. The current infection prevalence (35%, N = 23) is lower than that previously reported (60%, N = 25) in southwestern Québec, but is similar to the 37% (N = 191) prevalence reported in wolves from western and northern Canada in 2016 [10,15]. Although the cervid strain of *Echinococcus* was endemic to Québec in the 1980s, it should be noted that the molecular methods required to differentiate *E*. *canadensis* from *E*. *granulosus* did not exist at that time. Furthermore, increased sample sizes of wolves tested would improve the degree of confidence associated with prevalence estimates. Higher prevalence of *E*. *canadensis* in wolves versus coyotes might indicate that wolves predate upon intermediate hosts more frequently than coyotes. Known intermediate hosts for *E*. *canadensis* in Québec are moose, muskoxen (*Ovibos moschatus*) and caribou (*Rangifer tarandus*), but could reasonably also include elk (*Cervus canadensis*) and deer (*Odocoileus* spp.), as these have been reported elsewhere in Canada [8]. We did not detect *E*. *canadensis* north of Val d’Or. This is likely because we collected only foxes in northern Québec, and they are not considered common definitive hosts for *E*. *canadensis* (Fig 2) in comparison to wolves and coyotes. Surveillance of Inuit and Cree communities in the north of the province report sero-prevalence to echinococcosis (cystic or alveolar) ranging from 0.7% in James Bay to 8.3% in Nunavik [27,28], although it is unclear whether human cases have occurred. This suggests that infected wolves and/or coyotes continue to maintain the sylvatic lifecycle in northern Québec, and that people remain at risk of zoonotic transmission.

We did not detect *E*. *multilocularis* in Québec, despite sampling several potential definitive host species (coyotes, red/arctic foxes, wolves) within a few hundred kilometers of health regions in Ontario where infected canids were detected [3]. Although this distance is well within the migratory limits of such canids [29], it is possible that our availability sampling technique was not ideal for detection, as we collected no canids directly along the provincial border. An alternative explanation is that few canids are moving eastward from endemic hotspots in Ontario, due to the presence of three large urban centers (i.e. Ottawa, Montréal, and Sherbrooke) and their connecting roadways, or due to other geographic or ecological barriers. We believe our detection protocol was comprehensive, as it included morphological and molecular assessment of three *Echinococcus* spp. cestodes from each animal, which previously detected mixed *E*. *canadensis/E*. *multilocularis* infections in wolves [10], as well as an analysis of pooled samples of all *Echinococcus* spp. cestodes from each positive animal. Improved antemortem diagnostic tests for detecting and differentiating *Echinococcus* species in canids are needed to better determine prevalence and distribution, as *E*. *multilocularis* is emerging as a threat to human and animal health in North America.

Neither human nor animal echinococcosis cases are federally notifiable in Canada or the USA, although it recently became notifiable in Ontario, and surveillance of *Echinococcus* is outdated or nonexistent in some regions [1]. This negatively impacts efforts to track changes in *Echinococcus* distribution and incidence, or to accurately assess risks to animal and human health. Further work to characterize the geographic distribution, incidence, and health significance of these parasites across North America is warranted. Cross-border studies such as this one are important because emerging pathogens and their wildlife hosts do not observe political boundaries, and the movements of dogs across international borders is a known mechanism of spreading important zoonotic pathogens such as *Echinococcus*.

## References

[1] Cerda J, Buttke D, Ballweber L. *Echinococcus* spp. tapeworms in North America. Emerg Infect Dis. 2018;24:230–5. 10.3201/eid2402.161126 29350139 PMC5782903

[2] Lichtenwalner A, Adhikari N, Kantar L, Jenkins E, Schurer J. *Echinococcus granulosus* genotype G8 in Maine Moose (*Alces alces*). Alces. 2014;50:27–33.

[3] Kotwa J, Jardine C, Isaksson M, Berke O, Pearl D, Mercer N, et al Prevalence and geographic distribution of *Echinococcus multilocularis* in wild canids across southern Ontario, Canada In: Combating Zoonoses: Strength in East-West Partnerships [Internet]. Kuala Lumpur, Malaysia; 2017 [cited 2017 Nov 22]. Available from: https://miceapps.com/client/EventAttendeeAbstracts/view_published_abstract/305/4742/21766

[4] Romig T, Deplazes P, Jenkins D, Giraudoux P, Massolo A, Craig P, et al Ecology and life cycle patterns of *Echinococcus* species. Adv Parasitol. 2017;95:213–314. 10.1016/bs.apar.2016.11.002 28131364

[5] Eckert J, Gemmell M, Meslin F-X, Pawlowski Z. WHO/OIE Manual of echinococcosis in humans and animals: a public health problem of global concern Organization for Animal Health and World Health Organization; 2001.

[6] Deplazes P, Rinaldi L, Alvarez Rojas C, Torgerson P, Harandi M, Romig T, et al Global distribution of alveolar and cystic echinococcosis. Adv Parasitol. 2017;95:315–493. 10.1016/bs.apar.2016.11.001 28131365

[7] Santa M, Pastran S, Klein C, Duignan P, Ruckstuhl K, Romig T, et al Detecting co-infections of *Echinococcus multilocularis* and *Echinococcus canadensis* in coyotes and red foxes in Alberta, Canada using real-time PCR. Int J Parasitol Parasites Wildl. 2018; 10.1016/j.ijppaw.2018.03.001 PMC6031960 29988802

[8] Schurer J, Shury T, Leighton F, Jenkins E. Surveillance for *Echinococcus canadensis* genotypes in Canadian ungulates. Int J Parasitol Parasites Wildl. 2013;2:97–101. 10.1016/j.ijppaw.2013.02.004 24533321 PMC3862526

[9] Davidson R, Lavikainen A, Konyaev S, Schurer J, Miller A, Oksanen A, et al *Echinococcus* across the north: Current knowledge, future challenges. Food Waterborne Parasitol. 2016;4:39–53.

[10] Schurer J, Pawlik M, Huber A, Elkin B, Cluff D, Pongracz J, et al Intestinal parasites of gray wolves (*Canis lupus*) in northern and western Canada. Can J Zool. 2016;94:643–50.

[11] Trotz-Williams L, Mercer N, Walters J, Wallace D, Gottstein B, Osterman-Lind E, et al Public health follow-up of suspected exposure to *Echinococcus multilocularis* in southwestern Ontario. Zoonoses Public Health. 2017;64:460–7. 10.1111/zph.12326 28012251

[12] Peregrine A, Jenkins E, Barnes B, Johnson S, Polley L, Barker I, et al Alveolar hydatid disease (*Echinococcus multilocularis*) in the liver of a Canadian dog in British Columbia, a newly endemic region. Can Vet J. 2012;53:870–4. 23372195 PMC3398525

[13] Schurer J, Rafferty E, Farag M, Zeng W, Jenkins E. Echinococcosis: An economic evaluation of a veterinary public health intervention in rural Canada. PLOS Neglected Tropical Diseases. 2015;9. 10.1371/journal.pntd.0003883 PMC4489623 26135476

[14] Massolo A, Liccioli S, Budke C, Klein C. *Echinococcus multilocularis* in North America: the great unknown. Parasite. 2014;21:73 10.1051/parasite/2014069 25531581 PMC4273702

[15] McNeill M, Rau M, Messier F. Helminths of wolves (*Canis lupus* L.) from southwestern Quebec. Can J Zool. 1984;62:1659–60.

[16] Gesy K, Pawlik M, Kapronczai L, Wagner B, Elkin B, Schwantje H, et al An improved method for the extraction and quantification of adult *Echinococcus* from wildlife definitive hosts. Parasitol Res. 2013;112:1–4. 10.1007/s00436-013-3371-x 23471781

[17] Catalano S, Lejeune M, Liccioli S, Verocai G, Gesy K, Jenkins E, et al *Echinococcus multilocularis* in urban coyotes, Alberta, Canada. Emerg Infect Dis. 2012;18. 10.3201/eid.1810.120119 PMC3471618 23017505

[18] Bowles J, Blair D, McManus D. Genetic variants within the genus *Echinococcus* identified by mitochondrial DNA sequencing. Mol Biochem Parasitol. 1992;54:165–74. 1435857 10.1016/0166-6851(92)90109-w

[19] Bowles J, McManus D. NADH dehydrogenase 1 gene sequences compared for species and strains of the genus *Echinococcus*. Int J Parasitol. 1993;23:969–72. 8106191 10.1016/0020-7519(93)90065-7

[20] National Center for Biotechnology Information. Basic Local Alignment Search Tool [Internet]. [cited 2017 Nov 23]. Available from: https://blast.ncbi.nlm.nih.gov/Blast.cgi

[21] Trachsel D, Deplazes P, Mathis A. Identification of taeniid eggs in the faeces from carnivores based on multiplex PCR using targets in mitochondrial DNA. Parasitol. 2007;134:911–20. 10.1017/S0031182007002235 17288631

[22] R Core Team. R: A language and environment for statistical computing. [Internet]. Vienna, Austria; 2017. Available from: https://www.R-project.org/

[23] Nakao M, Yanagida T, Konyaev S, Lavikainen A, Odnokurtsev V, Zaikov V, et al Mitochondrial phylogeny of the genus *Echinococcus* (Cestoda: Taeniidae) with emphasis on relationships among *Echinococcus canadensis* genotypes. Parasitology. 2013;140(13):1625–36. 10.1017/S0031182013000565 23731519

[24] Carmena D, Cardona G. Echinococcosis in wild carnivorous species: Epidemiology, genotypic diversity, and implications for veterinary public health. Vet Parasitol. 2014;202:69–94. 10.1016/j.vetpar.2014.03.009 24698659

[25] Lichtenwalner A. Bulletin #1002, *Echinococcus granulosus canadensis* (EG) in Maine Moose: Suggestions for Dog Owners [Internet] University of Maine: Cooperative Extension Publications 2013 [cited 2018 Jan 23]. Available from: https://extension.umaine.edu/publications/1002e/

[26] Thompson R, Boxell A, Ralston B, Constantine C, Hobbs R, Shury T, et al Molecular and morphological characterization of *Echinococcus* in cervids from North America. Parasitol. 2006;132:439–47. 10.1017/S0031182005009170 16316488

[27] Messier V, Levesque B, Proulx J-F, Rochette L, Serhir B, Couillard M, et al Seroprevalence of seven zoonotic infections in Nunavik, Quebec (Canada). Zoonoses Public Health. 2012;59:107–17. 10.1111/j.1863-2378.2011.01424.x 21824376

[28] Sampasa-Kanyinga H, Levesque B, Anassour-Laouan-Sidi E, Cote S, Serhir B, Ward B, et al Zoonotic infections in native communities of James Bay, Canada. Vector-borne Zoonotic Dis. 2012;12:473–81. 10.1089/vbz.2011.0739 22217180

[29] Mech L, Boitani L. Canids, foxes, wolves, jackals and dogs. Status survey and conservation action plan [Internet] Sillero-Zubiri C, Hoffman M, Macdonald D, editors. IUCN/SSC Canid Specialist Group; 2004 [cited 2015 Nov 2]. Available from: http://www.env.gov.bc.ca/fw/wildlife/management-issues/docs/grey_wolf_management_plan.pdf

